# Giant invasive spinal schwannoma in children: a case report and review of the literature

**DOI:** 10.1186/1752-1947-7-289

**Published:** 2013-12-30

**Authors:** Sudhakar Vadivelu, Prashant Prasad, Adekunle M Adesina, Eugene Kim, Thomas G Luerssen, Andrew Jea

**Affiliations:** 1 Neuro-spine Program, Division of Pediatric Neurosurgery, Texas Children’s Hospital, Department of Neurosurgery, Baylor College of Medicine, 6621 Fannin Street, Houston, TX 77030, USA; 2 Division of Neuropathology, Texas Children’s Hospital, Department of Pathology, Baylor College of Medicine, 6621 Fannin Street, Houston, TX 77030, USA; 3 Division of Pediatric Surgery, Texas Children’s Hospital, 6621 Fannin Street, Houston, TX 77030, USA; 4 Present Address: Division of Pediatric Neurosurgery, Cincinnati Children’s Hospital Medical Center, 3333 Burnet Avenue, Cincinnati, OH 45229, USA

**Keywords:** Giant invasive spinal schwannoma, Pediatric spine, Reconstructions, Fusion

## Abstract

**Introduction:**

Giant invasive spinal schwannoma is defined as a tumor that extends over two or more vertebral levels, erodes vertebral bodies, and extends into the extraspinal space disrupting myofascial planes. Because of its rarity, there have been few published reports describing clinical features and surgical outcomes, especially in the pediatric patient population.

**Case presentation:**

We analyzed the medical record, pathologic findings, and radiographic studies of a 14-year-old Hispanic boy who presented to Texas Children’s Hospital with a three-month history of progressive spastic paraparesis. Preoperative computed tomography and magnetic resonance imaging reports showed a large mass lesion centered at the left T7-8 neural foramen with intra- and extraspinal extension, resulting in severe spinal cord compression and vertebral body erosion, and protrusion into the retropleural space and descending aorta. Our patient underwent a single-stage posterior approach for complete resection of the tumor with reconstruction and stabilization of the vertebral column. The pathological examination was consistent with schwannoma. At the six-month follow-up, our patient had resolution of preoperative symptoms and remains neurologically intact without any radiographic evidence of recurrent tumor.

**Conclusion:**

To the best of our knowledge, our case represents the fourth child with giant invasive spinal schwannoma reported in the literature. We describe our case and review the literature to discuss the aggregate clinical features, surgical strategies, and operative outcomes for giant invasive spinal schwannoma in the pediatric age group.

## Introduction

Schwannomas are benign tumors originating from Schwann cells, comprising of about 30% of primary intraspinal neoplasms [1]. They most commonly occur in nerve sheaths of the intradural extramedullary compartment. Most schwannomas are solid or mixed cystic-solid tumors, and can rarely undergo cystic degeneration, xanthomatous change, or hemorrhage [2,3]. Schwannomas are typically seen in adults between ages 40 and 60 years, and are rare in children. In a large referral center, only 0.7% of all schwannomas occurred in children during a 10-year period [4]. Pediatric schwannomas often occur in the setting of neurofibromatosis type 2 (NF-2).

Giant invasive spinal schwannomas are even more rare in the pediatric age group (<=18 years of age). These tumors present as huge masses that extend into the vertebral body and the extraspinal space as reported in case reports and small case series [5-12]. Complete removal of giant invasive spinal schwannomas is technically challenging due to its invasive nature, mass effect, and close proximity to important neurovascular structures [6,13]. The optimal surgical strategy for giant invasive spinal schwannomas involves decompression of the spinal cord and gross total resection, while preserving nearby neurovascular structures and addressing the potential for vertebral column instability. Reports on the clinical features of giant invasive spinal schwannomas and treatment approaches based on outcome are sparse [14-16]. Moreover, to the best of our knowledge, there have only been three previous descriptions of the clinical features of these tumor and surgical strategies in children [6,17]. We add a fourth case of giant invasive spinal schwannoma to the literature, analyze the clinical features and surgical outcomes in the context of previously reported cases, and discuss treatment strategies.

## Case presentation

A 14-year-old previously healthy Hispanic boy presented to the hospital with a two-month history of progressive worsening mid-back pain, bilateral leg pain, leg weakness, and unsteady gait. On examination, our patient had a spastic paraparesis with obvious signs of myelopathy, including lower extremity hyperreflexia, clonus, upgoing toes on Babinski response, impaired proprioception, and a T10 sensory level.

**Figure 1:**
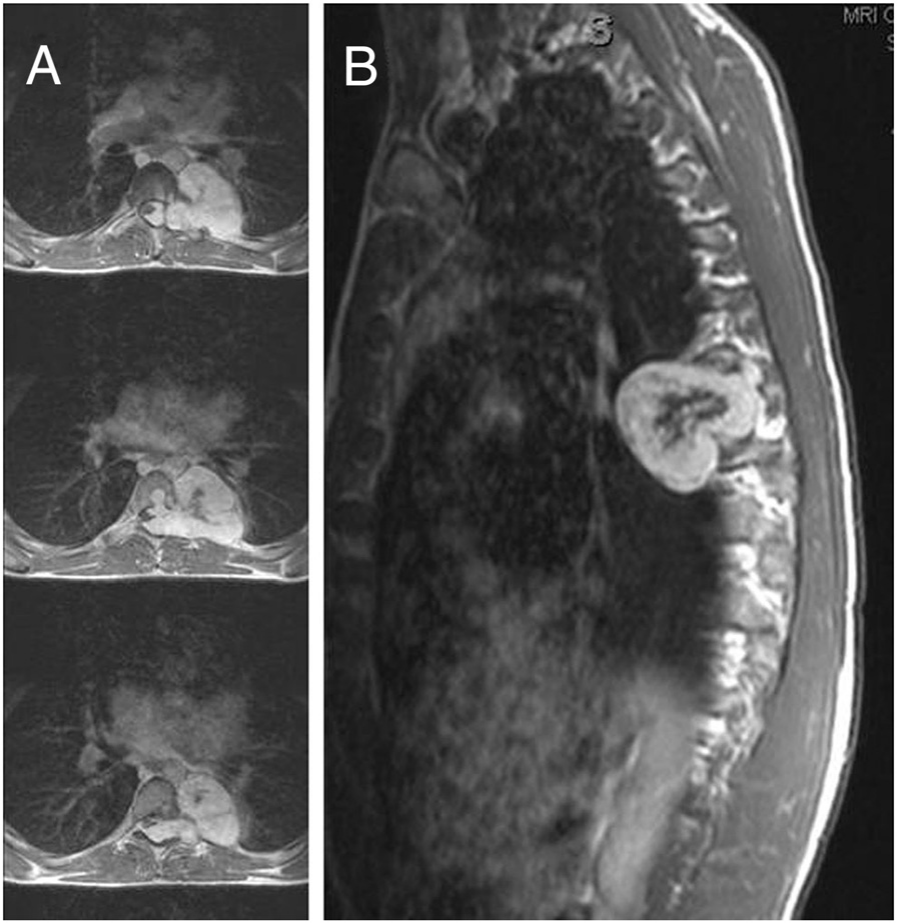
**Preoperative thoracic magnetic resonance imaging (MRI) scan demonstrates a giant invasive spinal schwannoma with spinal cord compression at T7-8 level. (A)** axial- and **(B)** sagittal-enhanced T1-weighted MRI scan also shows extension through the left T7-8 neural foramen into the retropleural space.

Our patient underwent a workup with a computed tomography (CT) scan of the thoracic spine without contrast with sagittal and coronal reconstructions, and a magnetic resonance imaging (MRI) scan of the thoracic spine with and without gadolinium. The CT scan showed a large left paraspinous soft tissue mass at T7, growing into and filling much of the spinal canal via an expanded and remodeled left T7-8 neural foramen. There was also focal lysis in the posterior T7 vertebral body as well as the left T7 pars, secondary to tumor erosion. The contrast-enhanced MRI scan demonstrated a 4×5×6.4cm heterogenous-enhancing mass with a partial hypointense center. The mass appeared with a classic dumbbell-shaped formation at the left T7-8 neural foramen with severe spinal cord compression (Figure 1).

Our patient was placed in a prone position for a lateral extracavitary approach for resecting this large spinal mass. Pre-incision intraoperative neuromonitoring parameters demonstrated no reproducible somatosensory-evoked (SSEPs) and motor-evoked potentials (MEPs) in the lower extremities. A standard subperiosteal dissection was performed from T6 to T8. Bilateral T6, T7, and T8 pedicle screws were placed, except at the left T7 pedicle. A temporary rod was secured on the right for stabilization. A laminectomy was then performed at these levels, and the tumor was observed exiting both the T7-8 neural foramen. Approximately 6cm of the 7th and 8th ribs were resected to complete the lateral extracavitary approach. The mass was mobilized after dissection using a soft tissue plane between the parietal pleura and extracanalicular portion of the tumor. The tumor was debulked and mobilized with the assistance of a Cavitron Ultrasonic Surgical Aspirator (CUSA). Once a surgical cavity was created laterally in the pleural cavity from tumor debulking, we mobilized and swept the portion of the tumor in the spinal canal medial-to-lateral away from the spinal cord into the surgical cavity, completing a gross total resection. The spinal construct was then completed including the use of a structural rib autograft spanning the bony defect created by resection of the facet joint, pars, and pedicle. At the end of the case, SSEPs remained baseline; however, MEPs were now present.

**Figure 2:**
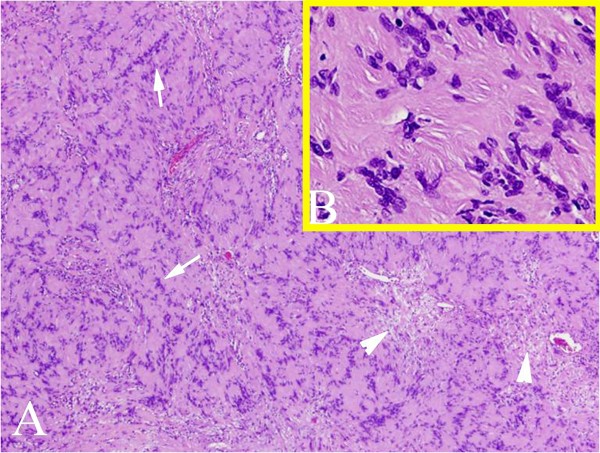
**Histology of paraspinal tumor showing a predominant Antoni type A pattern with florid formation of Verocay bodies (arrows).** Antoni type **B** regions are less prominent (arrow heads) Magnification **A**, ×40; **B**, inset ×400.

Hematoxylin and eosin staining demonstrated predominant Antoni type A pattern with florid formation of Verocay bodies in this spinal tumor. Antoni type B regions were observed as less prominent (Figure 2).

**Figure 3:**
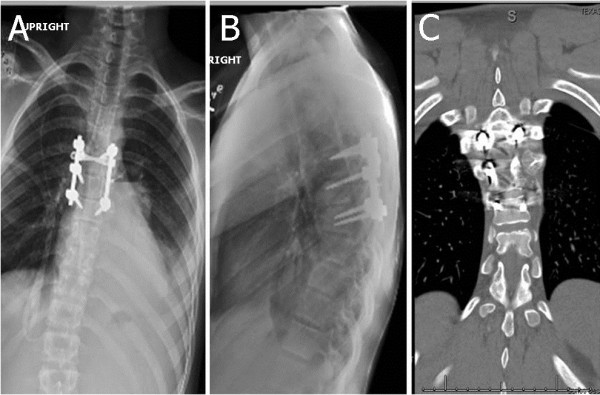
**Follow-up (A) anterior-posterior and (B) lateral X-rays, and (C) a coronal computed tomography (CT) scan at three months after surgery shows no evidence of recurrent or residual tumor.** There is evidence of bony healing without signs of instrumentation failure.

Postoperatively, our patient experienced slow improvement of his lower extremity weakness. Our patient was discharged home with a walker six days after surgery. At the six-month follow-up clinical visit, however, he was neurologically intact, and ambulating independently. Postoperative imaging at three months after surgery demonstrated no residual tumor, an evolving fusion mass, and stable posterior fusion construct (Figure 3).

## Discussion

Nerve sheath tumors of the spine are often single, small, benign lesions that are straightforward to remove and are associated with a good postoperative outcome. Giant invasive schwannomas, Type V by the classification of Sridhar *et al.*[6], differ from other giant schwannomas in that they erode the posterior surface of the vertebral body and may infiltrate through the dura and invade the surrounding myofascial planes while remaining histologically benign. Giant invasive spinal schwannomas have been reported infrequently in the pediatric age group [6,17] (Table 1). To the best of our knowledge, it has not been reported in the thoracic spine of a child.

**Table 1:** Patient demographics, operative details, and outcomes

**Case no.**	**Age (y), sex**	**Presentation**	**Tumor location**	**Procedure**	**Instrumentation for stabilization**	**Extent of resection**	**Ki-67 index, %**	**Postoperative course**	**Neurological outcome at last follow-up**	**Follow-up (months)**
**1**[15]	**15, F**	**Cauda equina syndrome, progressive b/l leg weakness**	**Lumbar (L2-5)**	**1**^**st**^**stage: laminectomy; 2**^**nd**^**stage: postero-lateral approach for tumor excision**	**none**	**GTR**	**N/A**	**Improved strength and daily brace**	**Normal motor strength and improved sensation BLE**	**18**
**2**[15]	**13, M**	**N/A**	**Lumbar**	**Laminectomy**	**none**	**N/A**	**N/A**	**N/A**	**N/A**	**N/A**
**3**[1]	**12, F**	**B/l leg pain and gait abnormality - 8 mos**	**Sacrum and pelvis**	**Laminectomy**	**N/A**	**GTR**	**N/A**	**N/A**	**N/A**	**N/A**
**4 (Current report)**	**14, M**	**Difficulty walking - 2 mos**	**Thoracic (T7, T8)**	**T6-T8 lateral extracavitary approach; T6-T8 posterior instrumented fusion**	**yes**	**GTR**	**<3%**	**Improved BLE strength and sensation**	**Normal strength and sensation**	**4**

BLE = bilateral lower extremities; GTR = gross total resection; N/A = not available.

Giant invasive spinal schwannomas may be encountered most frequently in the cervical spine [16] or lumbosacral and sacral spine [5,6]. In the present review of pediatric cases, the lumbar spine was most commonly affected (two cases), followed by the lumbosacral spine (one case), and thoracic spine (one case).

Gender predilection has not been determined in giant invasive spinal schwannomas. In our study, there were two males and two females. The average age of the patients in our study was 13 years (range, 12 to 15 years). The most common symptom in our study was pain (three of three patients), followed by motor weakness (two of three patients), and bowel and bladder symptoms (one of three patients). One of the previous studies reported limited clinical information.

Giant invasive spinal schwannomas makes conducting surgery technically challenging because of its growth in all directions and its sheer bulk and volume. They extend longitudinally over two or more levels; laterally into the extraspinal space through a widened and eroded foramen; posteriorly thinning the posterior elements of the vertebral column; and anteriorly eroding the vertebral bodies [5,6]. The approach, resectability, and stability of the spine are important preoperative considerations for the surgeon when facing this difficult tumor.

MRI is the primary neurodiagnostic tool in planning the surgical approach and predicting the resectability of giant invasive spinal schwannomas [6]. These tumors are heterogenous, with mixed hyperintensity and hypointensity on T2-weighted images corresponding to different cellularities, necrotic degeneration, hemorrhage, and cyst formation [5,18,19]. However, it may still be difficult to differentiate giant schwannomas from malignant peripheral nerve sheath tumors and ganglioneuromas based on imaging alone. The entire extent of the tumor may be seen in three dimensions. In addition, the relationship of the tumor to the spinal cord and/or nerve roots, vascular structures such as the great vessels, and other organs such as the lungs is delineated. A CT scan of the involved region of the vertebral column is mandatory in order to demonstrate the degree of bone destruction and to evaluate spinal stability [6].

Tumor eroding deep pockets into the vertebral bodies should be followed into bone and removed. The bone itself is devoid of tumor and therefore resection of bone for oncologic control is not warranted. Extraspinal extensions of these tumors are usually multilobulated. The surrounding tissues often form a pseudocapsule around the masses [6]. Occasionally, the paraspinal muscles may need to be divided - as in our case - to improve exposure of the pole of the extraspinal portion of the tumor.

Although we achieved a gross total resection of the giant invasive spinal schwannoma through a single stage and single approach, it may not always be possible to remove the entire tumor in one operative sitting. In these situations, a combined or multi-stage approach may be necessary [5]. The spine surgeon and patient must be prepared for multiple operations before achieving complete resection.

The pathological findings and growth potential for giant invasive spinal schwannomas has not been determined. The majority of patients have typical pathological findings of nerve sheath tumors (Figure 3). Ki-67 is expressed during the proliferative phase of the cell cycle and has been used to predict tumor regrowth after removal. It could be especially useful for predicting tumor growth in cases when total resection is not attained. The Ki-67 in our study was less than 3%.

It is important to plan and predict the need for reconstruction and stabilization of the vertebral column once a significant portion of the tumor has been removed [6]. Erosion of the vertebral body in combination with loss of the posterior elements is likely to render the vertebral column incompetent. In fact, even prior to the surgical removal of bony elements to expose the tumor, we advocate the placement of spinal instrumentation on the contralateral side of the approach to provide a reassuring degree of spinal stability while tumor resection occurs. Erosion of more than 25% of the vertebral body requires some form of reconstructive or stabilizing procedure [6]. Still, some surgeons have preferred not to fuse or stabilize the spine.

The use of spinal instrumentation does carry significant disadvantages. The most important disadvantage is the casting of the metal artifact on follow-up imaging for tumor surveillance. This is less of a concern in the case of complete tumor resection where tumor recurrence rate is low. However, in cases where partial or subtotal resection is performed, spinal instrumentation may make it easier to determine if there has been small tumor growth on subsequent CTs or MRIs.

The results of complete resections of giant invasive spinal schwannomas have been positive [5,6]. Most patients in individual case reports and small case series do well [5-12]. Recurrences should be managed with repeat surgeries [5,6]. The goal of each surgery should be complete resection of the mass. The role of adjuvant therapy has not been defined for this disease entity.

In the present study, gross total resection was achieved in three of three patients (operative details were not provided for one of the four patients). Of the patients undergoing gross total resection, one patient underwent a second operation for tumor resection as the first operation consisted only of a posterior decompression without any attempt at tumor resection.

## Conclusions

Giant invasive spinal schwannomas are uncommon tumors and distinct from other schwannomas. The accumulated clinical experience for these tumors in the pediatric age group is virtually nonexistent. We sought to report our own experience in a single case and to review the literature for other pediatric cases. It is clear that because of their local aggressiveness and extension in an omidirectional manner, careful preoperative planning including determination of surgical approach, assessment of resectability, and preparedness to restabilize the vertebral column is important. Complete resection of giant invasive spinal schwannomas is possible with good outcomes; however, they have a tendency to recur, necessitating close follow-up and possible repeat surgeries.

## Consent

Written informed consent was obtained from the patient’s legal guardian for publication of this manuscript and any accompanying images. A copy of the written consent is available for review by the Editor-in-Chief of this journal.

## Abbreviations

CT: Computed tomography; CUSA: Cavitron ultrasonic surgical aspirator; MEP: Motor-evoked potential; MRI: Magnetic resonance imaging; SSEP: Somatosensory-evoked potential.

## Competing interests

The authors declare that they have no competing interests.

## Authors’ contributions

SV, PP, and AJ analyzed the patient data and medical records, and were major contributors in writing the manuscript. AMA performed the histological examination of the tumor, and provided pathologic micrographs to support the manuscript. EK and TGL critically reviewed and revised the manuscript. All authors read and approved the final manuscript.
